# Dynamic assessment of global longitudinal strain after isometric exercise to predict functionally significant coronary lesion

**DOI:** 10.1093/ehjimp/qyag007

**Published:** 2026-01-12

**Authors:** Niya Mileva, Dobrin Vassilev, Panayot Panayotov, Svetlin Tsonev, Slawomir Golebiewski, Gianluca Rigatelli, Robert J Gil

**Affiliations:** Cardiology Department, Medica Cor Hospital, Riga 35, Ruse 7013, Bulgaria; Medical University of Pleven, Pleven, Bulgaria; Cardiology Department, Medica Cor Hospital, Riga 35, Ruse 7013, Bulgaria; Department of Public Health and Healthcare, Ruse University ‘Angel Kanchev’, Ruse, Bulgaria; Cardiology Department, Medica Cor Hospital, Riga 35, Ruse 7013, Bulgaria; Medical University of Pleven, Pleven, Bulgaria; Cardiology Department, Aleksandrovska University Hospital, Sofia, Bulgaria; Department of Interventional Cardiology and Internal Diseases, Military National Research Institute, Zegrzynska 8 Str, Legionowo, Poland; Cardiology Department, Ospedali Riuniti Padova Sud, Padova, Italy; Cardiology Department, National Medical Institute of Internal Affairs and Administration Ministry, Warsaw, Poland

**Keywords:** chronic coronary syndrome, non-invasive stress test, speckle tracking

## Abstract

**Aims:**

There is still a wide debate regarding the management of patients with chronic coronary syndrome, the choice of optimal non-invasive stress test, and the decision for coronary revascularization. Evidence has accumulated demonstrating the utility of global longitudinal strain (GLS) as a predictor of significant coronary disease. To evaluate the application of speckle tracking during resting echocardiography and after isometric loading with the hand-grip test, and to compare GLS parameters with fractional flow reserve (FFR).

**Methods and results:**

Patients with known CCS who underwent angiography with evidence of coronary stenosis >40%<90% and were referred for functional assessment were enrolled in this study. Patients with previous MI, depressed LV systolic function, and poor acoustic window were excluded. Patients underwent a standard echocardiography with recordings suitable for LV speckle tracking. Subsequently, a dynamic hand-grip test was performed for 3 min, then the same images were captured. Global longitudinal strain was calculated at rest (rGLS), under stress conditions (sGLS), as well as the absolute difference between the two measurements – ΔGLS. Then, patients underwent invasive functional assessment with measurement of the FFR of the coronary lesion. Correlation analysis of myocardial strain parameters and FFR was performed. A total of 106 patients were included in the study. The mean rGLS was −18.5 ± 1.8, sGLS: −19.1 ± 2.0 and ΔGLS: 0.62 ± 1.4. In total, 44% of patients had a functionally significant coronary lesion when assessed with FFR. There was a statistically significant correlation between ΔGLS and FFR values, r = 0.660, *P* < 0.001. ΔGLS of 0.35 showed good diagnostic performance capacity (AUC 0.896, 95% CI: 0.83–0.96, specificity 85%, sensitivity 92%) for a functionally significant coronary lesion.

**Conclusion:**

Dynamic GLS during isometric hand-grip exercise is a feasible method for functional assessment in patients with chronic coronary syndrome.

## Introduction

Chronic coronary syndrome (CCS) remains a leading cause of mortality and morbidity worldwide, affecting millions of people globally.^1^ The management of patients with CCS requires a combination of medical therapy with or without coronary revascularization.^2^ Contemporary clinical recommendations advise on the application of non-invasive techniques as the initial diagnostic approach for the optimal risk stratification of patients with a working diagnosis of CCS.^2,3^ The choice of the non-invasive method should be based on the pre-test likelihood of obstructive coronary artery disease (CAD), as well as specific patient characteristics, local expertise, and availability.^2^ Although several non-invasive methods have been introduced for the evaluation of ischaemia presence in patients with CAD, data point to their varying diagnostic reliability when compared to invasive functional assessment of coronary lesion severity.^4^ The advantages of stress echocardiography include its wide availability, low cost, and the lack of ionizing radiation. Pharmacologic stress echocardiography is preferred over exercise ECG in patients with suspected CAD but is time-consuming, operator-dependent, and requires a certain level of expertise to achieve accurate and reproducible results.^5^ Additionally, there is a growing body of evidence showing that the assessment of myocardial deformation by speckle tracking techniques provides additional information on regional and global left ventricular (LV) function and increases the reproducibility of the results.^6,7^ Global longitudinal strain (GLS) is a parameter that expresses longitudinal shortening and has been proven as a strong predictor of CAD as well as other cardiac pathologies.^8–11^ Measuring LV deformation by GLS has been shown to be a reproducible and sensitive measure of ischaemia in patients with CCS that may detect ischaemia with high accuracy compared to visual assessment.^12,13^ In fact, data reveals that adding GLS measurements to the protocol of pharmacological stress echo increases the diagnostic precision.^14,15^

Isometric hand-grip exercise is a simple method that was introduced to augment cardiac workload more than 30 years ago.^16^ The main response to a hand-grip exercise is an increase in systolic blood pressure and myocardial wall stress. It is a low-cost, easily accessible and widespread method. Hand-grip has previously been used alone, as well as in combination with other stress agents, for the detection of CAD.^17–19^ This isometric hand-grip exercise may be easily applied to the standard echocardiographic protocol with speckle tracking in patients with CCS.

Isometric hand-grip exercise induces myocardial ischaemia through distinct mechanisms compared to pharmacologic stress. Hand-grip increases myocardial oxygen demand primarily through elevated systolic blood pressure and myocardial wall stress, while simultaneously causing coronary vasoconstriction via sympathetic nervous reflex, thereby decreasing myocardial oxygen supply.^20–22^ This dual mechanism makes handgrip particularly suitable for detecting endothelial dysfunction and microvascular disease.^21^ While hand-grip alone has modest sensitivity (only 3–26% in early studies),^22^ combining it with speckle tracking GLS assessment addresses this limitation. When added to dobutamine stress echocardiography, hand-grip improved ischaemia detection from 62% to 83% (*P* < 0.05) by increasing left ventricular wall stress.^19^ Our protocol leverages this synergy by using GLS, which is more sensitive than visual wall motion assessment for detecting subtle ischemic changes. The advantages of our approach include: (1) avoidance of pharmacologic agents and their contraindications/side effects, (2) simplicity and low cost, (3) wide applicability in resource-limited settings, and (4) feasibility in patients who cannot exercise on a treadmill but can perform hand-grip.

We aimed to evaluate the diagnostic accuracy of a hand-grip stress echocardiography with speckle tracking assessment and compare it with invasive functional assessment with fractional flow reserve (FFR) in patients with CCS and intermediate coronary lesions.

## Materials and methods

### Study design and population

This is a prospective observational study designed to assess the application of speckle tracking during resting echocardiography and after isometric loading with the hand-grip test and to compare myocardial strain parameters with the gold standard for invasive functional assessment of coronary stenosis- fractional flow reserve (FFR). The study group was composed of consecutive patients admitted with chronic coronary syndrome and referred for invasive coronary angiography from January 2023 to September 2024 at the Specialized Cardiology Hospital, Medica Cor, Ruse, Bulgaria. These patients were screened to identify those who (i) had a previously identified >40% and ≤90% coronary stenosis [either by coronary computed tomography (CT) angiography or invasive angiography]; and (ii) had echocardiography with good quality images with ECG trace and the apical four-chamber, two-chamber, and three-chamber views available for speckle-tracking assessment. Exclusion criteria were reduced LV systolic function, presence of LV myocardial aneurysm or scar, presence of significant heart block, acute coronary syndrome within 6 months, known cardiomyopathy, and patients with poor acoustic window.

### Ethics

All patients were managed following the Declaration of Helsinki and provided informed consent for anonymous publication of scientific data. All authors have read and approved the final version of the manuscript and have no conflict of interest to declare in relation to the present work. The study protocol was approved by the local ethics committee (18/15.12.2023).

### Echocardiography protocol

All transthoracic echocardiograms were performed using a high-quality ultrasound machine (Philips, Affinity 7 and Philips EPIQ 11) with a 5–1 MHz-phased array transducer (X5-1c). All patients had a comprehensive 2D echocardiographic assessment with the recommendations of the European Association of Cardiovascular Imaging with the subjects in the left lateral recumbent position with the use of standard parasternal and apical views.^23^ All images were stored for offline analysis. LV ejection fraction (LVEF) was calculated using Simpson’s biplane method. Longitudinal myocardial deformation was assessed using the automated 2D speckle tracking technique (Philips, AutoStrain) in the three apical views with a typical temporal resolution of 60–90 frames/s. The regional speckle area of interest was manually adjusted to obtain optimal tracking results. LV GLS was calculated using a 17-segment model at the time in systole when the value peaked. In patients with atrial fibrillation, we selected loops from the apical four-chamber, two-chamber, and three-chamber views with comparable R-R intervals for strain calculation. The analysis of the images recorded was performed by two expert echocardiography cardiologists, blinded to clinical information. The GLS at rest (rGLS), under stress conditions (sGLS), and the absolute difference between the two measurements (ΔGLS) were calculated. GLS values are reported as negative numbers by convention, with more negative values indicating greater longitudinal shortening. For the calculation of ΔGLS, we used the formula: ΔGLS = sGLS—rGLS. Since both values are negative, a positive ΔGLS indicates worsening (less negative) strain with stress, while a negative ΔGLS indicates improvement (more negative) strain with stress. Absolute values were not used in our calculations.

### Isometric hand-grip exercise protocol

**Figure 1:**
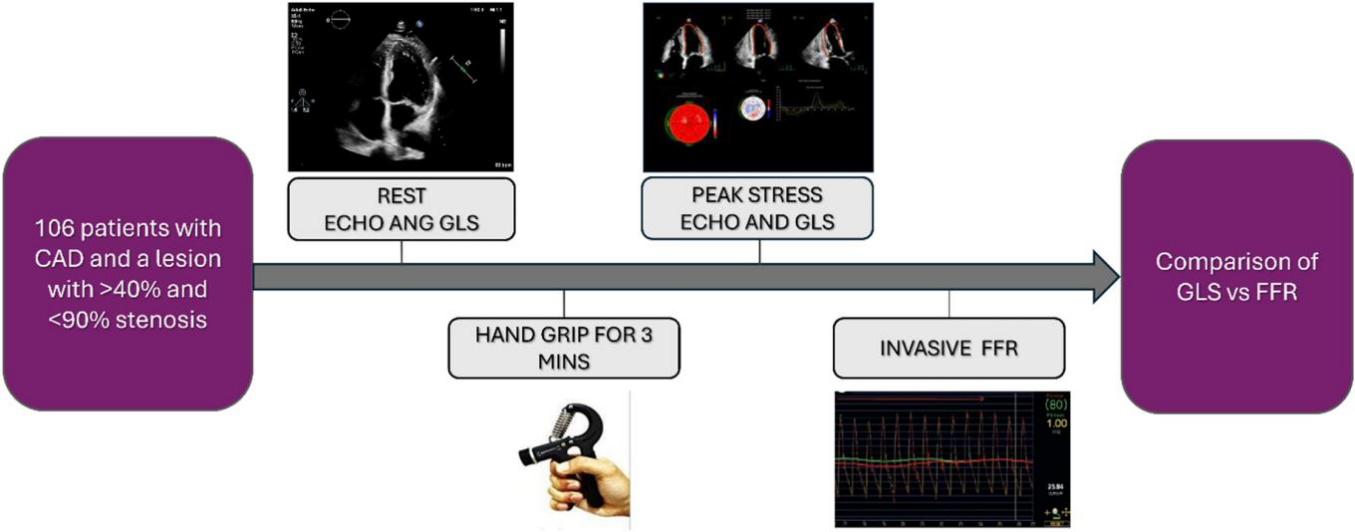
Study protocol. CAD – coronary artery disease; ECHO – echocardiography; GLS – global longitudinal strain; MINS – minutes; FFR—fractional flow reserve.

After obtaining the comprehensive TTE at rest, the participant stayed in a left lateral recumbent position, asked to hold a dynamometer with their right hand with the wrist in a neutral position.^24,25^ A calibrated hydraulic hand dynamometer (Jamar, Patterson Medical) was used for all measurements. Maximum voluntary contraction was first determined by having patients perform three maximal grip attempts with 30-second rest intervals, and the highest value was recorded. The target force (30% of maximum) was marked on the dynamometer, and patients received real-time visual feedback to maintain consistent force throughout the 3-minute exercise period. Participants were then instructed to grip the dynamometer with 30% of a perceived maximum voluntary contraction for 3 min. (*Figure 1*) Heart rate and blood pressure were continuously monitored during hand-grip exercise.

### Coronary angiography and fractional flow reserve measurements

Invasive coronary angiography was performed following local protocols in the participating hospitals. FFR measurements were performed following the recommendations of the Standardization of Fractional Flow Reserve Measurements document.^26^ Coronary angiography was performed through either radial or femoral arterial access. A 6 Fr guiding catheter was used, and 0.2 mg of intracoronary isosorbide dinitrate was administered. A guidewire equipped with a pressure/temperature sensor (PressureWire X; Abbott) was connected to dedicated software for tracing analysis (CoroFlow Cardiovascular System; Coroventis) and, after zeroing, was advanced through the guiding catheter. The pressure wire sensor was positioned in distal coronary segments >2 mm in diameter by visual estimation. Hyperaemia was induced through administration of intracoronary adenosine or papaverine, based on current availability and the operator's discretion, as both agents achieve maximal hyperaemia when used at appropriate doses.^27^ FFR was defined as the lowest ratio between distal and proximal coronary pressures during hyperaemia. FFR ≤ 0.80 was defined as abnormal.

### Sample size calculation

To determine the appropriate sample size for this prospective study, we utilized a power analysis based on the primary outcome of interest. We aimed for a significance level (α) of 0.05 and a power (1—β) of 0.80, which are standard thresholds in clinical research. Using previous literature, we estimated a Pearson correlation coefficient between GLS and FFR of 0.3. Given these parameters, we calculated that a total of 103 participants would be required to detect a statistically significant difference between the groups. Ultimately, we included 106 patients in the study to ensure that we met our target sample size, which allows for robust statistical analyses and increases the reliability of our findings.

### Statistical analyses

The normal distribution of continuous variables was assessed visually with histograms and with Shapiro–Wilk test. Continuous variables were summarized using the median and interquartile range. Categorical variables are presented as frequency counts and percentages. Fisher’s exact test was used for comparing categorical variables, while Mann–Whitney test for continuous ones. One-way ANOVA or Kruskal-Wallis tests were performed to test the difference of continuous variables between more than two groups, when appropriate. Correlation analysis of myocardial strain parameters (rGLS, sGLS, and ΔGLS) and FFR was assessed by Spearman’s method. The diagnostic performance of each echocardiographic speckle tracking parameter for the prediction of functionally significant lesions (abnormal FFR) was evaluated by receiver-operating characteristic (ROC) analyses. The optimal cut-off value was defined as the value that maximizes the sum of sensitivity and specificity using the Youden test. Positive predictive value (PPV), and negative predictive value (NPV) were calculated by standard formulas. A *P* value < 0.05 was considered statistically significant. Intra- and inter-observer reproducibility analysis on a subset of 30 randomly selected patients (representing 28% of the cohort) was performed. Two independent observers, blinded to FFR results and each other’s measurements, analysed rGLS, sGLS, and ΔGLS. All analyses were performed using the Statistical Package for Social Sciences, version 25.0 (SPSS, PC version, Chicago, IL, USA) and R Studio version 2024.09.1 + 394 (R Foundation for Statistical Computing, Vienna, Austria).

## Results

### Overall population

From January 2023 to September 2024, 161 patients with chronic coronary syndrome and moderate coronary artery lesions were referred to the study. Of these, 44 patients had a suboptimal acoustic window, 11 patients had an LV aneurysm or scar interfering the LV strain analysis, and were considered ineligible for the study. Therefore, the final population consisted of 106 patients, included in the study. The mean age of the population was 66.4 ± 9.8 years, with most of the patients being male (69%). 104 (98%) of them had hypertension, 31 (29%) patients had diabetes, and 45 (42.5%) of the population had a history of smoking. Detailed baseline demographic and clinical characteristics of the whole population are listed in *Table 1*.

**Table 1:** Demographic and clinical characteristics

Patient characteristics	All *n* = 106	FFR ≤ 0.80 *n* = 47	FFR > 0.80 *n* = 59	*P*-value
Age, years (mean ± sd)	66.4 ± 9.8	65.9 ± 9.4	66.7 ± 10.2	0.223
Males, n (%)	73 (68.9)	35 (74.5)	38 (64.4)	**0**.**019**
BMI, mean ± sd	27.9 ± 4.5	28.9 ± 4.8	27.0 ± 4.2	0.264
Hypertension, n (%)	102 (96.8)	45 (95.7)	57 (96.6)	0.573
Dyslipidemia, n (%)	100 (94.3)	44 (93.6)	56 (94.9)	0.512
Diabetes, n (%)	31 (29.2)	17 (36.2)	14 (23.7)	**0**.**023**
Chronic kidney disease, n (%)	11 (10.4)	5 (10.6)	6 (10.2)	0.817
Cancer, n (%)	5 (4.7)	2 (4.3)	3 (5.1)	0.104
Smoking, n (%)	45 (42.5)	20 (42.6)	25 (42.4)	0.673
Atrial fibrillation, n (%)	13 (12.3)	12 (25.5)	9 (15.3)	**0**.**030**
Cerebrovascular disease, n (%)	8 (10.9)	3 (11.1)	4 (10.2)	0.149
Peripheral artery disease, n (%)	5 (4.7)	3 (6.4)	2 (3.4)	**0**.**040**
COPD, n (%)	12 (11.3)	7 (14.9)	5 (8.5)	**0**.**028**

FFR – fractional flow reserve; BMI – body mass index; COPD – chronic obstructive pulmonary disease. Bolded values indicating significant values.

The mean increase in systolic blood pressure was 28 ± 12 mmHg (from 132 ± 18 to 160 ± 22 mmHg, *P* < 0.001), and the mean heart rate increased by 18 ± 8 bpm (from 72 ± 12 to 90 ± 14 bpm, *P* < 0.001). All patients achieved at least a 15 mmHg increase in systolic blood pressure, confirming adequate hemodynamic stress. All patients performed hand-grip with their dominant hand (right hand in 94% of patients). In the 6% who were left-handed, the left hand was used.

Coronary lesion was located in the left anterior descending artery (LAD) in 71 (67%) of the patients. In 18 patients (17%) target lesion was the left circumflex artery (LCx), whereas in 17 patients (16%), the artery of interest was the right coronary artery (RCA). The averaged values of measured myocardial strain parameters were rGLS: −18.5 ± 1.8, sGLS: −19.1 ± 2.0, and ΔGLS: 0.62 ± 1.4.

### Difference between groups with functionally significant and non-significant lesions

The mean measured FFR of the whole study cohort was 0.81 ± 0.07. Hyperaemia was induced using either intracoronary adenosine (20–40 μg) in 64% of patients or intracoronary papaverine (20 mg) in 36%, based on operator preference and local availability. In total, 47 (44%) patients had a functionally significant coronary lesion when assessed with FFR (≤0.80), and 59 patients (56%) had a functionally nonsignificant lesion with an FFR >0.80. When comparing the two groups the cohort with functionally significant lesions were more frequently male (75% vs. 64%, *P* = 0.019), had more diabetes (36% vs. 24%, *P* = 0.023), more atrial fibrillation (25% vs. 15%, *P* = 0.030), more peripheral artery disease (6% vs. 3%, *P* = 0.040) and more COPD 14.9% vs. 8.5%, *P* = 0.028), *Table 1*.

### Standard echocardiographic findings and speckle tracking parameters

Evaluation of traditional echocardiographic parameters did not reveal any significant difference between the two groups. (*Table 2*, Supplementary data online, *Figure S1*). A comparison of speckle tracking indices between the two cohorts showed that there was no significant difference between the values of GLS at rest. Patients with functionally significant coronary lesions based on FFR had numerically higher GLS (−18.0 ± 1.82 vs. −20.1 ± 1.72, *P* = 0.105) when compared to patients with functionally non-significant lesions. The only statistically significant parameter that differed between the two groups was the ΔGLS: −0.39 ± 0.78 vs. 1.43 ± 1.22, *P* < 0.001, in the functionally significant vs. functionally nonsignificant lesions, respectively.

**Table 2:** Echocardiographic and angiographic characteristics

Patient characteristics	All *n* = 106	FFR ≤ 0.80 *n* = 47	FFR > 0.80 *n* = 59	*P*-value
LV ejection fraction, mean ± sd	57.9 ± 5.31	58.4 ± 5.35	57.6 ± 5.31	0.224
Mitral regurgitation ≥2 grade, n (%)	39 (36.8)	18 (38.3)	21 (35.6)	0.065
E/e’ medial (mean ± sd)	12.7 ± 5.02	10.0 ± 2.44	14.8 ± 5.52	0.092
LA volume index, mL/m2 (mean ± sd)	28.6 ± 6.33	28.9 ± 4.79	27.7 ± 6.81	0.410
rGLS, mean ± sd	−18.5 ± 1.75	−18.4 ± 1.80	−18.6 ± 1.73	0.762
sGLS, mean ± sd	−19.1 ± 2.03	−18.0 ± 1.82	−20.1 ± 1.72	0.105
ΔGLS, mean ± sd	0.62 ± 1.38	−0.39 ± 0.78	1.43 ± 1.22	**<0**.**001**
Previous PCI, n (%)				
Target vessel, n (%)				
LAD	71 (66.9)			
LCx	18 (17.0)			
RCA	17 (16.1)			

FFR – fractional flow reserve; LV – left ventricle; LA – left atrium; GLS – global longitudinal strain; PCI – percutaneous coronary intervention; LAD – left anterior descending artery; LCx – left circumflex artery; RCA – right coronary artery.

### Reproducibility analysis

We have conducted post-hoc reproducibility analysis on a subset of 30 randomly selected patients (representing 28% of the cohort). Two independent observers, blinded to FFR results and each other’s measurements, analysed rGLS, sGLS, and ΔGLS. Intraobserver variability (same observer, 2 weeks apart) revealed ICC 0.89 (95% CI 0.81–0.94) for ΔGLS. Interobserver variability was characterized with ICC 0.85 (95% CI 0.74–0.92) for ΔGLS. Coefficient of variation 8.2% for ΔGLS.

### Association between GLS and invasive FFR

**Figure 2:**
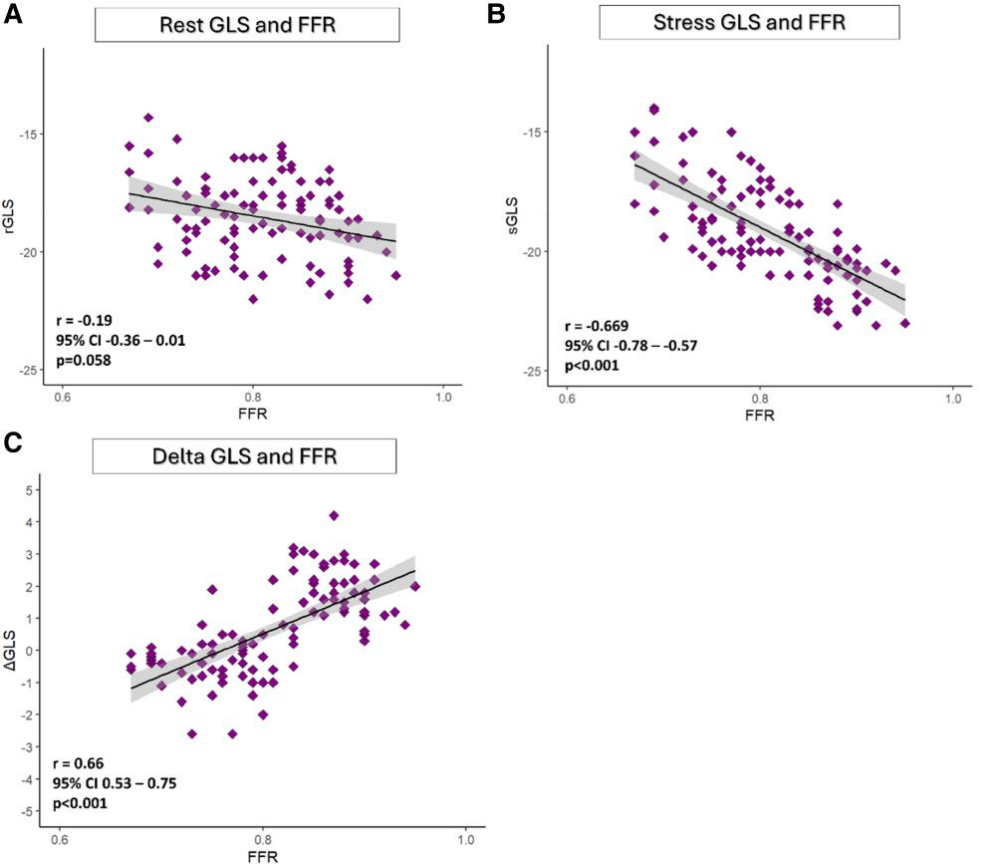
Correlation plots. (*A*) GLS at rest and FFR; (*B*) GLS at peak stress and FFR; (*C*) Delta GLS (absolute difference between stress and rest GLS) and FFR. GLS – global longitudinal strain; FFR – fractional flow reserve; r – Pearson correlation coefficient; CI – confidence intervals.

There was a moderate correlation between the values of GLS at rest and FFR values, but no statistically significant correlation, r = −0.19, 95%CI −0.36—−0.01, *P* = 0.058 (*Figure 2*, panel A). There was a statistically significant correlation between sGLS and FFR—r = −0.669, 95%CI −0.78—−0.57, *P* < 0.001, (*Figure 2*, panel B), and ΔGLS and FFR values, and r = 0.660, 95%CI 0.53–0.75, *P* < 0.001 (*Figure 2*, panel C), respectively. We have performed a subgroup analysis comparing FFR values obtained with adenosine (*n* = 68) vs. papaverine (*n* = 38): mean FFR with adenosine was 0.81 ± 0.07 vs. mean FFR with papaverine: 0.80 ± 0.08, *P* = 0.42. The correlation between ΔGLS and FFR was similar in both subgroups: Adenosine subgroup: r = 0.64, *P* < 0.001 vs. papaverine subgroup: r = 0.68, *P* < 0.001, *P* for interaction = 0.58.

### Predictive role and diagnostic performance of GLS stress test for patients with functionally positive coronary lesions

**Figure 3:**
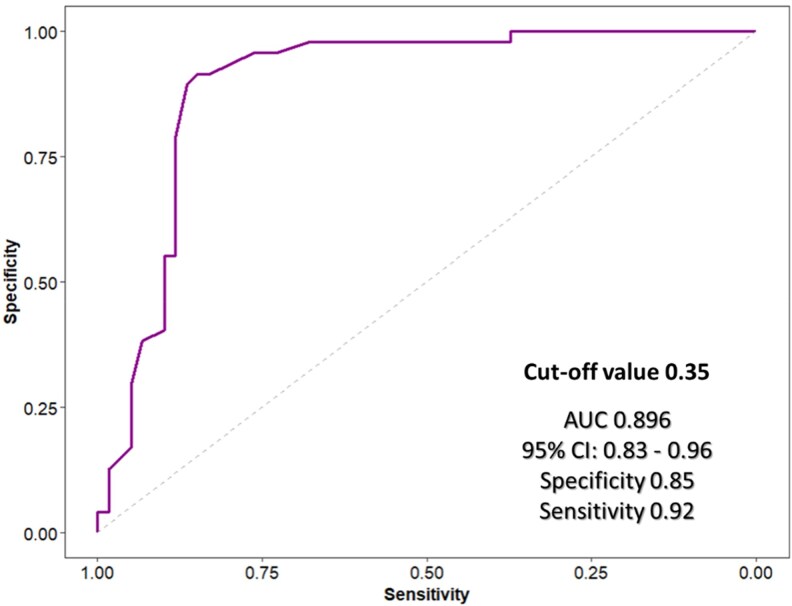
Predictive capacity of delta GLS to detect functionally significant coronary lesions based on FFR. GLS – global longitudinal strain; FFR – fractional flow reserve; AUC – area under the curve; CI – confidence intervals.

**Figure 4:**
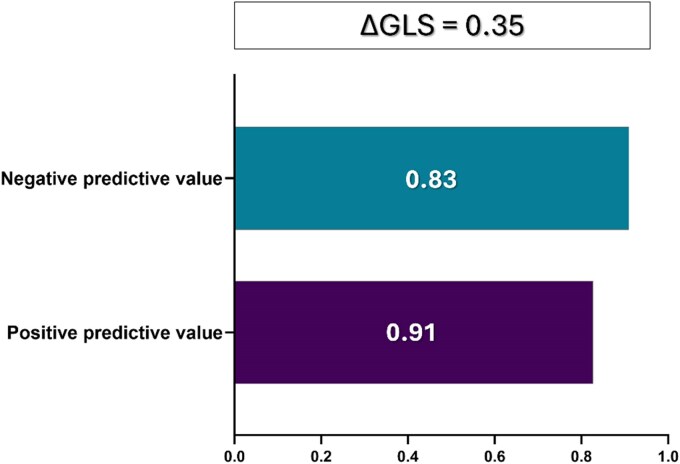
Barplot visualization of the negative and positive predictive value of the delta GLS of 0.35 to predict a functionally significant lesion. ΔGLS – Delta GLS (absolute difference between stress and rest global longitudinal strain).

The ROC analysis revealed that, among the different speckle tracking indices, ΔGLS has the best diagnostic performance (AUC = 0.896, 95% CI = 0.832–0.961; *P* < 0.001, *Figure 3*). The optimal cut-off of ΔGLS was 0.35 with 92% of sensitivity 85% of specificity. Based on this value, nine patients were defined as positive on the GLS stress test but were negative when tested with FFR. On the contrary, only four patients who were identified as negative based on the GLS stress test were found positive with the invasive FFR. The positive predictive value (PPV) of the GLS stress test was 0.827. The negative predictive value (NPV) of the test is 0.910. (*Figure 4*)

## Discussion

To the best of our knowledge, the present study is the first one to describe a protocol of isometric hand-grip exercise combined with speckle tracking assessment – the GLS isometric stress test. In fact, the diagnostic performance of isometric exercise echocardiography with speckle tracking assessment has not been studied in relation to angiography with functional assessment until now. The main findings of our study are: i) The study introduces a simple novel stress protocol of easily applicable technique for the evaluation of patients with CCS and intermediate coronary lesions; ii) The GLS index that demonstrated best correlation with FFR was the change in GLS with isometric exercise (ΔGLS); iii) A cut-off value of ΔGLS of 0.35 had excellent predictive capacity with AUC 0.896, 92% sensitivity, and 85% specificity to define patients with functionally positive coronary lesions. This single-centre study provides proof-of-concept that dynamic GLS assessment during isometric hand-grip is feasible and shows promising diagnostic performance.

Based on previously published data, current clinical practice guidelines suggest that CCS patients without high-risk features should be referred to an initial strategy of OMT and lifestyle modification.^2,3^ Coronary revascularization may be retained for the patients whose symptoms are refractory on medical therapy, with risk remaining that an unfavourable event, including sudden cardiac death (1.3% in ISCHAEMIA trial) may occur.^28^ However, the timing of when to offer coronary revascularization has remained an immense dispute. While contemporary recommendations have focused on OMT, there are wide disparities in how the guidelines are interpreted and applied between specialties, institutions, and countries.

The ISCHAEMIA trial was conducted to resolve the equipoise regarding the efficacy of early revascularization plus OMT compared to OMT alone in patients with CCS and non-invasive evidence of myocardial ischaemia.^29^ The main message of the trial appeared to be that an invasive strategy was no better than OMT for the reduction of cardiovascular events in patients with CCS. Although this headline suggests the conclusion of the debate, questions remain on whether the study design was the most appropriate approach. There are also concerns on whether the selected cohort represents the wider population of patients with CCS and if the most appropriate non-invasive testing was applied. The ISCHEMIA trial highlighted ongoing debate regarding optimal patient selection for revascularization. Non-invasive functional testing remains central to this decision-making. Our protocol offers potential advantages in settings where pharmacologic stress is contraindicated or unavailable, though its role relative to established modalities requires further study.

Hand-grip has previously been used alone, as well as in combination with other stress agents, for the detection of CAD.^17–19^ The induction of ischaemia during hand-grip is believed to be due to an increase in myocardial oxygen demand resulting from an increase in blood pressure and heart rate. In addition, hand-grip has also been shown to cause coronary vasoconstriction due to a sympathetic nervous reflex, thereby decreasing myocardial oxygen supply.^30^ Used alone, hand-grip is only moderately sensitive for detecting CAD.^30,31^ The major mechanism of the increase in heart rate with hand-grip is a centrally mediated vagal withdrawal.^32^ Adding speckle tracking assessment to the isometric hand-grip exercise may increase the diagnostic accuracy of the method. The assessment of LV deformation through speckle tracking and GLS calculation has been shown to be a reproducible and sensitive measure of ischaemia in patients with CCS. A study demonstrates that LV deformation measured by GLS normally increases during physiologic stress and remains unchanged or reduces in the ischaemic myocardium.^15^

However, GLS normally varies with age, sex, LV loading conditions, and even different software.^33^ Therefore, defining a specific value of an abnormal GLS may not be straightforward. Nevertheless, the absolute change in GLS with application of myocardial stress is a parameter that may act as a marker with higher accuracy of myocardial ischaemic changes. In fact, our study revealed that ΔGLS correlated much strongly with FFR than the rest and stress GLS by their own. An absolute difference of 0.35 GLS before and after stress was able to achieve 92% sensitivity and 85% specificity for the detection of patients with functionally significant coronary lesions. There was a weak correlation between resting GLS and FFR that did not reach statistical significance (r = −0.19, 95% CI −0.36 to −0.01, *P* = 0.058), suggesting that resting strain measurements alone are insufficient for functional assessment.

Another important aspect to consider is that a substantial proportion of the patients with CCS present without any significant coronary obstruction on coronary angiogram.^34^ The current diagnosis of patients with angina (ANOCA) or ischaemia with non-obstructive coronary arteries (INOCA) and its different endtypes is mainly determined by invasive coronary functional testing.^2^ Further research is needed to refine and assess non-invasive diagnostic imaging modalities for patients with coronary microvascular dysfunction. It is unclear how the isometric stress GLS would be changed in patients with ANOCA/INOCA spectrum.

### Limitations

Despite the important findings being mentioned, several limitations of the study need to be recognized. The single-centre observational study design represents a methodological limitation with respect to the applicability of the study results. Multicentre research on a larger scale is necessary to support these findings. The exclusion of 34% of screened patients, primarily due to poor acoustic windows (27%), represents a limitation that may affect generalizability. However, this exclusion rate is comparable to other stress echocardiography studies and reflects the real-world challenge that speckle tracking requires adequate image quality.^35,36^ The use of contrast echocardiography, which we did not employ in this study, could potentially reduce this exclusion rate in future investigations. Importantly, patients with poor acoustic windows would also be excluded from standard stress echocardiography protocols, suggesting our findings remain applicable to the population suitable for any echo-based stress testing.

While we have now provided reproducibility data from a subset analysis, prospective validation of the reproducibility of this specific handgrip-GLS protocol in a larger cohort would strengthen confidence in the methodology. Our findings are in agreement with previous studies that showed high agreement between different observers and measurements of GLS.^37,38^ The small magnitude of the ΔGLS cut-off (0.35) requires careful quality control and may be susceptible to measurement variability, emphasizing the need for standardized protocols and adequate training. In the acknowledgement of these limitations, the study results remain therefore hypothesis generating. Multicentre validation studies are needed before this approach can be recommended for routine clinical use.

## Conclusion

Dynamic speckle tracking testing during isometric hand-grip exercise is a feasible method for functional assessment in patients with chronic coronary syndrome. The absolute difference of GLS during stress and rest showed excellent correlation with FFR, and the cut-off value of ΔGLS 0.35 had excellent predictive capacity to define patients with functionally positive coronary lesions.

## Supplementary Material

qyag007_Supplementary_Data

## Data Availability

The data that support the findings of this study are available on request from the corresponding author, NM.

## Lead author biography

Dr Niya Mileva is a cardiologist and cardiovascular imaging specialist based in Ruse, Bulgaria. She graduated with an MD from the Medical University of Sofia in 2016, completing specialist cardiology training there. This was followed by a Cardiovascular Imaging Fellowship at OLV Cardiovascular Centre in Aalst, Belgium (2020–2022), and cardiac magnetic resonance training at Cardiovascular Institute Paris Sud in 2023. Dr Mileva holds EACVI certifications in adult transoesophageal echocardiography and cardiac computed tomography, as well as passing the European Exam in Core Cardiology. She has a PhD degree from the Medical University of Sofia and is an active researcher with 59 publications, a Scopus h–index of 18 and over 1,000 citations. As a Fellow of the European Society of Cardiology and a committee member of Heart Imagers of Tomorrow, she combines clinical care, advanced imaging and research to improve cardiovascular diagnosis and treatment.
