# Supplementary material for: Dynamic assessment of global longitudinal strain after isometric exercise to predict functionally significant coronary lesion
Source: Eur Heart J Imaging Methods Pract. 2026 Jan 12;4(1):qyag007. doi: 10.1093/ehjimp/qyag007 (PMC12871075; doi:10.1093/ehjimp/qyag007)
Supplement: qyag007_Supplementary_Data [file qyag007_supplementary_data.docx]

**Dynamic Assessment of Global Longitudinal Strain after Isometric Exercise to Predict Functionally Significant Coronary Lesion**

**Supplementary Material**

**Supplementary figure 1***.* A) distribution plot of GLS at rest; B) distribution plot of GLS at stress; C) distribution plot of delta GLS.

*
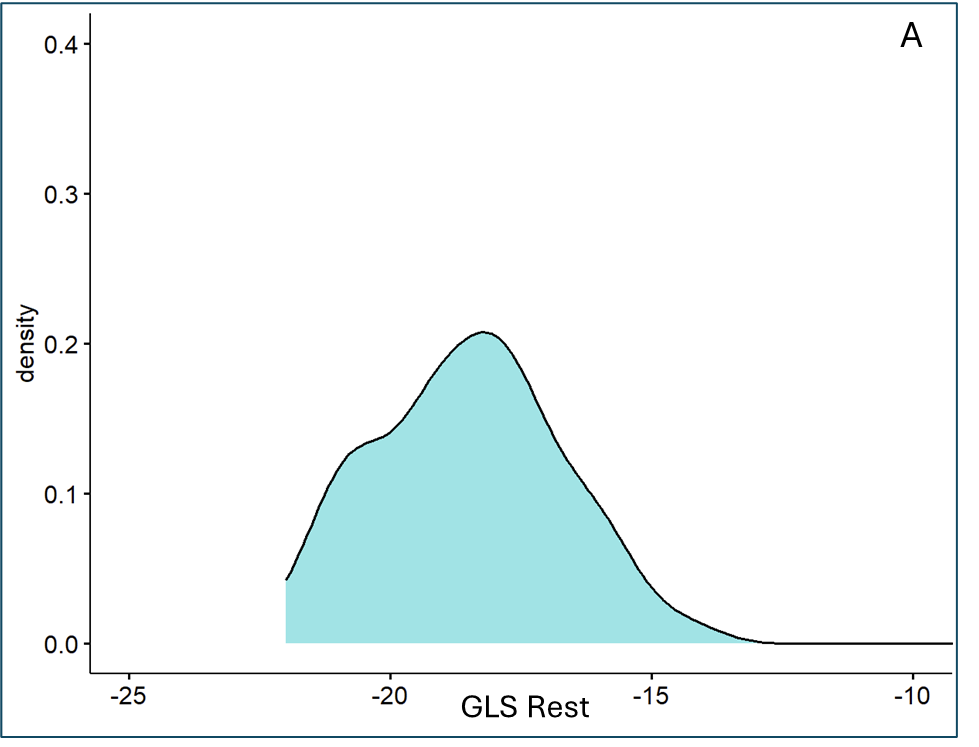
*

*
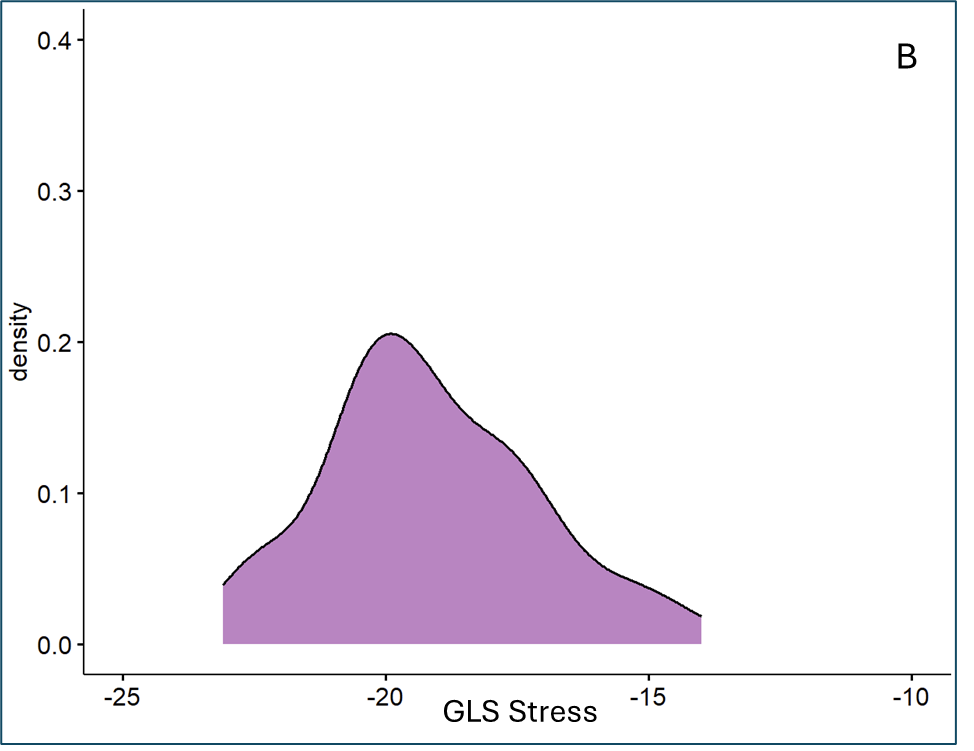
*

*
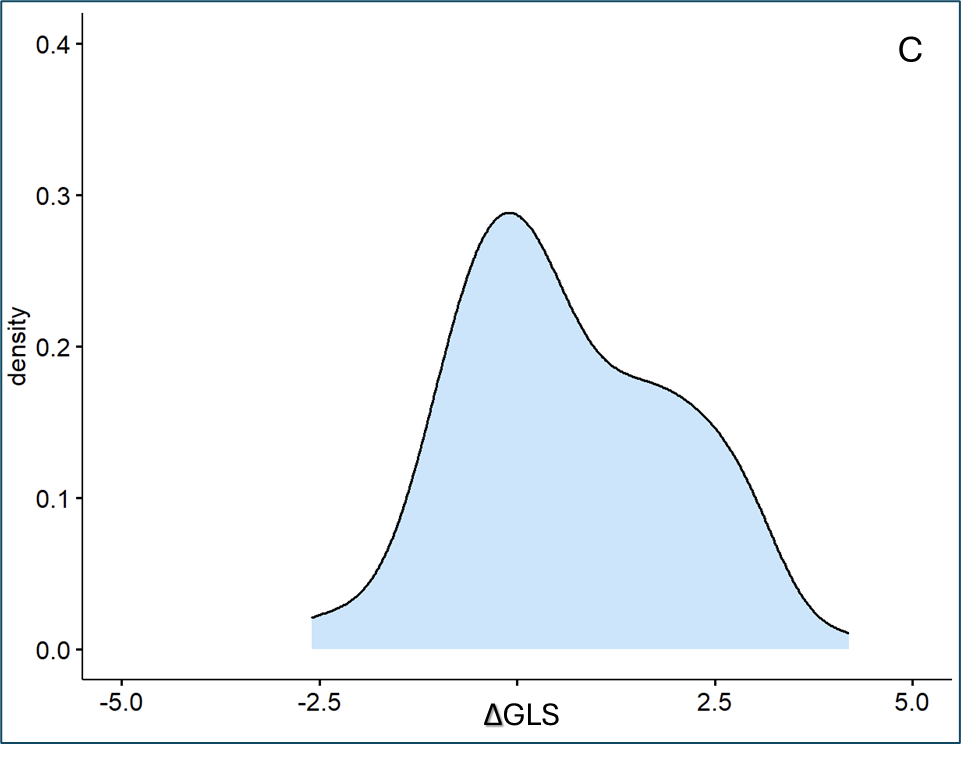
*
